# Antibacterial effect of phage cocktails and phage-antibiotic synergy against pathogenic *Klebsiella pneumoniae*

**DOI:** 10.1128/msystems.00607-24

**Published:** 2024-08-21

**Authors:** Mengshi zhao, Hongru Li, Dehao Gan, Mengzhu Wang, Hui Deng, Qiu E. Yang

**Affiliations:** 1 College of Resources and Environment, Fujian Agriculture and Forestry University, Fuzhou, China; 2 Fujian Key Laboratory of Traditional Chinese Veterinary Medicine and Animal Health, Fujian Agriculture and Forestry University, Fuzhou, China; 3 Department of Infectious Disease, Shengli Medical College, Fujian Medical University, Fujian Provincial Hospital, Fuzhou University affiliated Provincial Hospital,, Fuzhou, China; University of Illinois at Chicago, Chicago, Illinois, USA

**Keywords:** carbapenem-resistant *Klebsiella pneumoniae*, phage therapy, phage-antibiotic synergy, multidrug resistance

## Abstract

The global rise of antibiotic resistance has renewed interest in phage therapy, as an alternative to antibiotics to eliminate multidrug-resistant (MDR) bacterial pathogens. However, optimizing the broad-spectrum efficacy of phage therapy remains a challenge. In this study, we addressed this issue by employing strategies to improve antimicrobial efficacy of phage therapy against MDR *Klebsiella pneumoniae* strains, which are notorious for their resistance to conventional antibiotics. This includes the selection of broad host range phages, optimization of phage formulation, and combinations with last-resort antibiotics. Our findings unveil that having a broad host range was a dominant trait of isolated phages, and increasing phage numbers in combination with antibiotics significantly enhanced the suppression of bacterial growth. The decreased incidence of bacterial infection was explained by a reduction in pathogen density and emergence of bacterial resistance. Furthermore, phage-antibiotic synergy (PAS) demonstrated considerable broad-spectrum antibacterial potential against different clades of clinical MDR *K. pneumoniae* pathogens. The improved treatment outcomes of optimized PAS were also evident in a murine model, where mice receiving optimized PAS therapy demonstrated a reduced bacterial burden in mouse tissues. Taken together, these findings offer an important development in optimizing PAS therapy and its efficacy in the elimination of MDR *K. pneumoniae* pathogens.

**IMPORTANCE:**

The worldwide spread of antimicrobial resistance (AMR) has posed a great challenge to global public health. Phage therapy has become a promising alternative against difficult-to-treat pathogens. One important goal of this study was to optimize the therapeutic efficiency of phage-antibiotic combinations, known as phage-antibiotic synergy (PAS). Through comprehensive analysis of the phenotypic and genotypic characteristics of a large number of CRKp-specific phages, we developed a systematic model for phage cocktail combinations. Crucially, our finding demonstrated that PAS treatments not only enhance the bactericidal effects of colistin and tigecycline against multidrug-resistant (MDR) *K. pneumoniae* strains in *in vitro* and *in vivo* context but also provide a robust response when antibiotics fail. Overall, the optimized PAS therapy demonstrates considerable potential in combating diverse *K. pneumoniae* pathogens, highlighting its relevance as a strategy to mitigate antibiotic resistance threats effectively.

## INTRODUCTION

Antimicrobial resistance (AMR) poses a major threat to human health around the world, which has been claimed to cause over 10 million deaths annually by 2050 (1). *Klebsiella pneumoniae* is emerging as one of the six most notorious pathogens contributing to the global AMR burden in causing over 929,000 (660,000–1270,000) deaths in 2019 (2). In this context, the combination of carbapenem resistance and pathogen *K. pneumoniae* (CRKp) keeps emerging as a major cause of mortality in hospital settings (3, 4), which responsible for 50,000–100,000 deaths in 2019 alone. Those CRKp strains are generally associated with the production of carbapenmase, notably KPC-2 and NDM-1 producers (5, 6), which confer resistance to nearly all currently available antibiotics including non-β-lactam antibiotics. The alarmingly rapid emergence of CRKp strains seems to outpace our discovery and development of new antibiotics; therefore, the current AMR situation displays an urgent need to develop novel antibiotic-alternative strategies to fight against CRKp strains.

The idea of using bacteriophages, a century-old antibacterial therapeutic agents, has been renewed lately (7). Phages are viruses that specifically infect bacterial cells, leading to the lysis of the bacterial host. Phage therapy provides a promising complement to antibiotics against multidrug-resistant (MDR) bacterial infections (8–10), not only due to phage being the most abundant biological entity on Earth but also a range of intrinsic differences in their bactericidal mechanisms. Preclinical studies of phage therapy in mouse models have demonstrated encouraging results in successfully suppressing *K. pneumoniae* bacteremia (9, 11–13). Furthermore, clinical case studies have established the contribution of phage therapy in treating patients with severe wound infections (14), prosthetic joint infections (15), inflammatory bowel diseases (9), and urinary tract infections (16).

The use of phage in combination with antibiotics has been proposed to combat MDR pathogens and delay the evolution of phage resistance. The logic of combining phages and antibiotics is that two distinctively different bactericidal mechanisms are likely to be more effective than either alone. A prime example of this is the so-called phage-antibiotic synergy (PAS), which means that when antibiotics are combined with phages, they can potentially trigger a synergistic effect in suppressing the growth of bacterial pathogens (17). Importantly, besides enhancing the suppression of bacterial pathogens, the PAS treatment also has been shown to reduce the formation of biofilm and prevent the emergence of resistant variants (18). For instance, phage combined with ciprofloxacin or gentamicin showed promising antibiofilm activity, leading to the successful elimination of the pathogens *Pseudomonas aeruginosa* and *Staphylococcus aureus*, whereas antibiotics or phage alone only had a modest effect in reducing biofilm bacteria (19, 20). It is of note that PAS is highly dependent on the mechanism of bacterial inhibition by the class of antibiotics (21), and the underlying mechanisms of this synergy with antibiotics remain unclear.

Despite the fact that PAS seems to be a promising alternative to compensate for the failure of antibiotics, most, if not all, studies displayed its efficacy only on one specific strain. In order to expand the bactericidal spectrum of PAS, we aim to find phages with the broadest possible host ranges and ultimately search for optimal “one size fits all” phage-antibiotic combinations. Herein, we applied a host-cocktail enrichment method to isolate 100 *K*. *pneumoniae* phages, the majority of which exerted broad-range activity toward 88 MDR Kp strains. Due to their MDR profiles, phage or antibiotic monotherapy had a modest effect in the suppression of most bacterial growth. In contrast, when applied simultaneously, the PAS combinations, phage cocktail P7 with colistin in particular, achieved synergistic, rather than just additive effects against most of MDR Kp strains (*n* = 66/88, 75%). The optimized PAS therapy significantly decreased the emergence of phage resistance in a murine model, resulting in enhanced efficacy of antibiotic-phage combinations in the suppression of bacterial growth. Overall, our findings validate the selection of broad-host range phages and enable an effective and broad-spectrum PAS treatment against CRKp strains *in vitro* and *in vivo*.

## RESULTS

### Antibiotic resistance of MDR clinical *K. pneumoniae* strains

**Fig 1:**
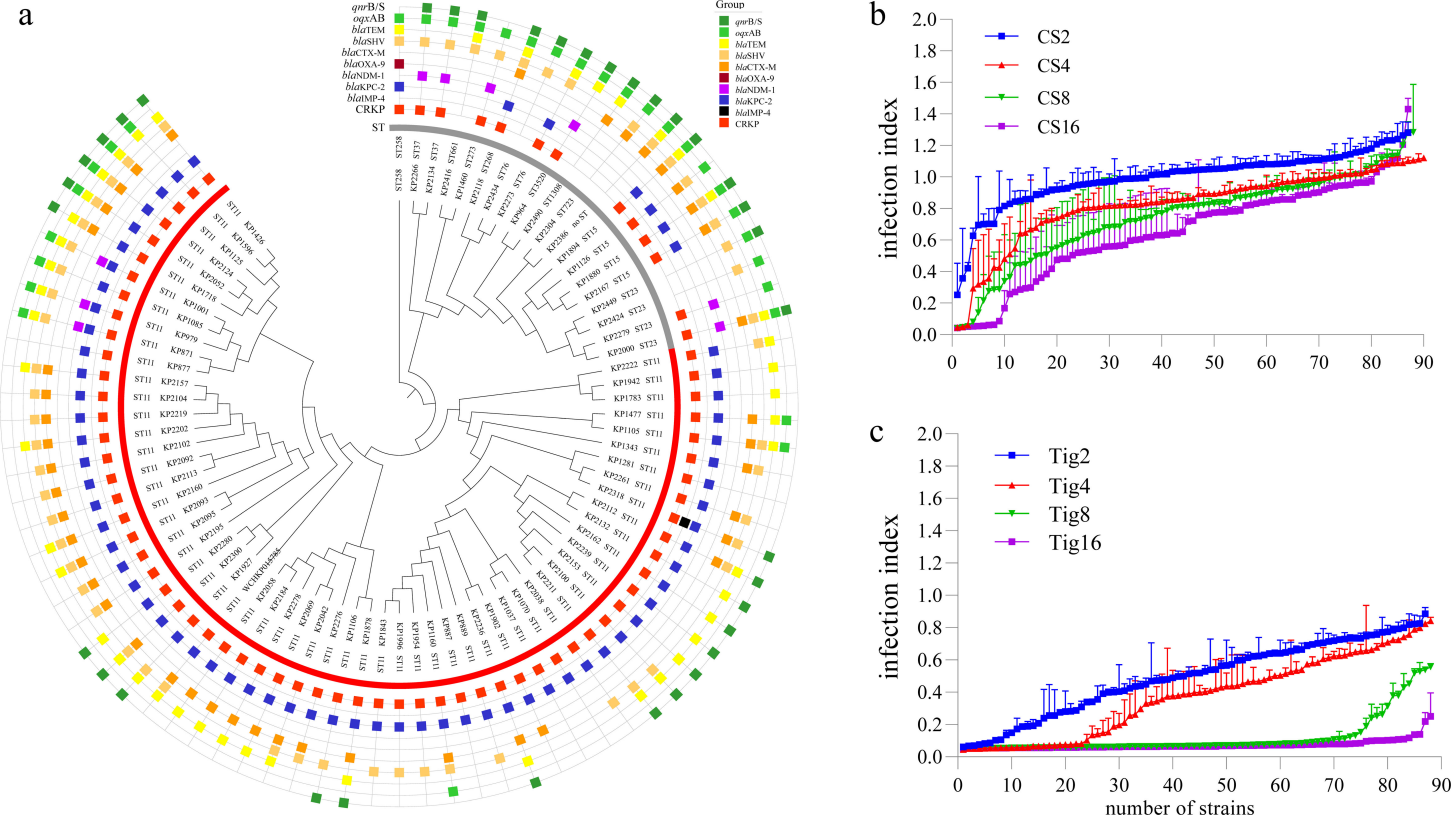
(a) Phylogenic analysis of 80 MDR *K. pneumoniae* strains based on whole genome sequencing. Each branch in the phylogenetic tree represents one strain, with sequence type (ST) and resistance genes shown in different colored squares outside the circle. Eight MDR *K. pneumoniae* strains from this collection did not generate a tree. Two reference *K. pneumoniae* genomes included in this tree were strain WCHKP015785 (ST11, GeneBank No. JAEMHP010000000) and strain ST258 (ST258, GeneBank No. LGAB01000000). (b**-c**) *In vitro* antimicrobial activity of antibiotic-alone (colistin or tigecycline) with different concentrations including the 4 mg/L of clinical dose against 88 MDR kp strains, indicating that antibiotic monotherapy can effectively suppress a few numbers of MDR Kp strains. CS2/4/8/16 indicates the mono-antibiotic therapy with 2, 4, 8, or 16 mg/L colistin, respectively; Tig2/4/8/16 indicates the mono-antibiotic therapy with 2, 4, 8, or 16 mg/L tigecycline, respectively. The plotted values represent mean ± SEM (*n* = 3).

A total of 88 MDR *K. pneumoniae* (Kp) isolates were retrieved from patients in Fujian Provincial Hospital (China), and subsequently, whole genome sequencing (WGS) and minimal inhibitory concentration (MIC) analysis were performed. The obtained results confirmed that the majority of the tested isolates belong to sequence type ST11 (*n* = 61), known for its prevalence in nosocomial *K. pneumoniae* infections and its extensive drug-resistant profiles (Fig. 1a; Data S1). WGS and subsequent PCR results revealed that 80 of the isolates harbored either *bla*_KPC_ (*n* = 70) or *bla*_NDM_ (*n* = 10) genes coding for carbapenemase, which is a common feature of this high-risk CRKp ST11 clone. Additionally, WGS analysis showed that each tested strain carried 6 to 17 genetic determinants known to confer antimicrobial resistance, which was associated with their extensive drug-resistance phenotype showing insensitivity to almost all β-lactams including meropenem, imipenem, ceftazidime, ceftriaxone, and cefoxitin, as they only remained susceptible to the last-resort antibiotics, colistin and tigecycline (MIC ≤2 mg/L). However, clinical treatment doses (4 mg/L, Fig. 1b and c) of colistin or tigecycline were only able to suppress a few strains (bacterial infection index <0.2, *n* = 3 for colistin and *n* = 30 for tigecycline), indicative of likely therapeutic failure. However, higher concentrations (8 and 16 mg/L) of tigecycline can effectively suppress the majority of MDR Kp strains.

### Broad host range of the isolated *Klebsiella*-specific phages

**Fig 2:**
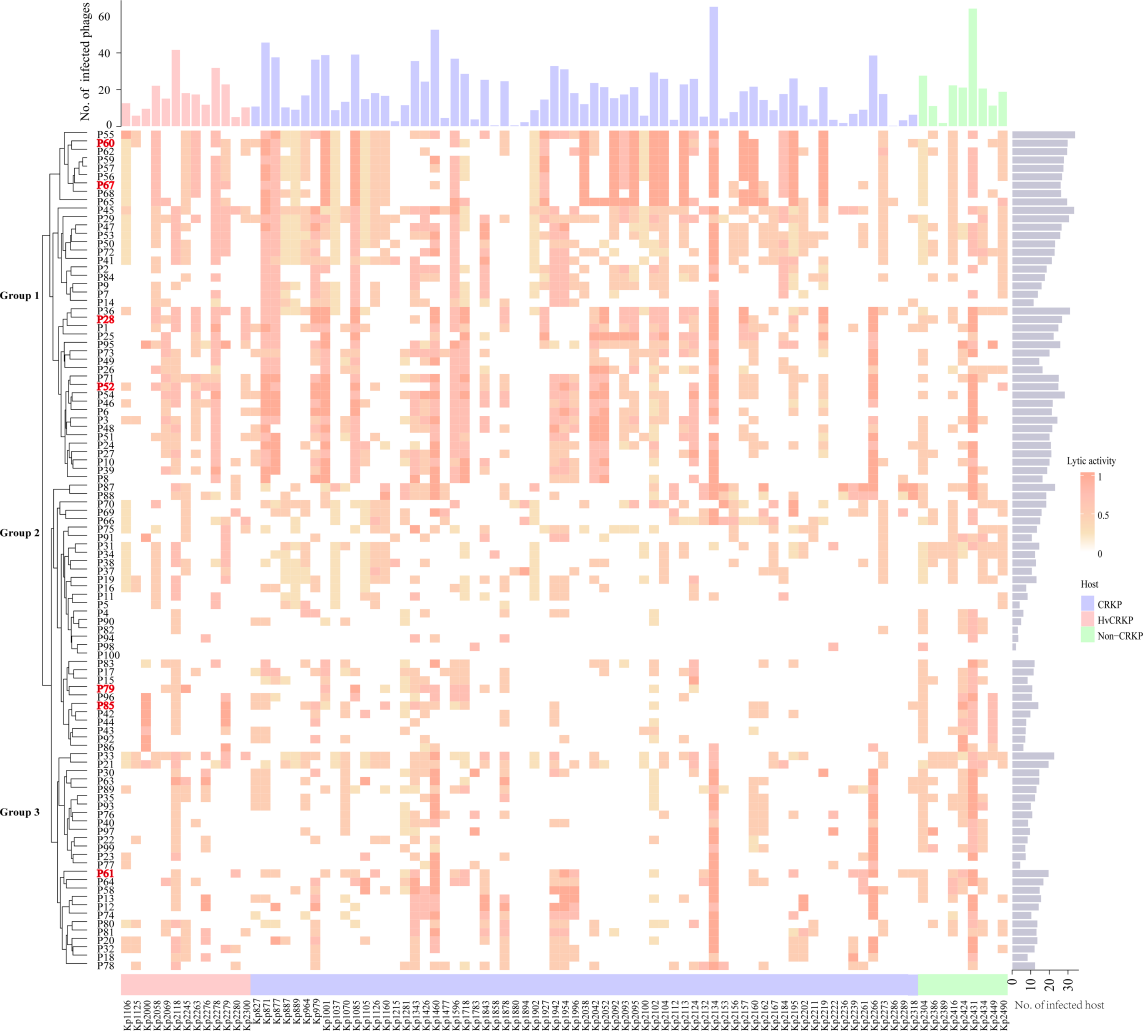
A heat map representing the *Klebsiella* phages host range against 88 MDR *K. pneumoniae* strains. Phage susceptibility was assessed using a spot-test assay, and the clarity and transparency of the plaques observed after spot assay were scored as 1, 0.75, 0.5, 0.25, or 0 (see methods for details and data set 1). The clustering process was carried out on the basis of phage host ranges and their infectivity profiles/scores. The brighter shade of the red square suggests greater phage-inhibiting activity, as indicated by the color scale on the right side of the heat map. The bar chart to the right side of the heat map shows the number of Kp strains that each phage can infect. Meanwhile, the bar chart above the heat map indicates the number of phages that can infect each Kp strain. The CRKp and non-CRKp strains were distinguished by different colors. Seven phages were highlighted in red, as they were selected for the combination of phage cocktails in Fig. 3a.

Given the failure of monotreatment by clinical doses of antibiotics (4 mg/L, Fig. 1b and c), we sought to develop a PAS approach that specifically suppressed the pathogenic CRKp clades isolated from patients. Initially, we aimed to isolate broad host range phages targeting 88 MDR Kp strains by applying an enrichment process with cocktails of multiple strains (10 strains per cocktail) that were designed to produce a diverse bacterial community. Using those bacterial cocktails to enrich broad host range phages, a total of 100 phages were isolated and purified using a double-layer agar plate method (Fig. 2).

To discriminate all isolated phages and unravel host ranges, the double-layer plaque method was applied by challenging a diverse host panel of 88 clinical *K. pneumoniae* strains (Fig. 1a). A heat map was generated on the basis of the phage host ranges and their infectivity profiles (Fig. 2; Data S1). Through hierarchical clustering of phage infection profiles, these phages were collapsed into three major cluster groups, indicating the diversity of phage-host interactions. We observed a remarkably high level of infectivity from group 1 (Fig. 2), as most phages showed broad host ranges with 36.9 strains on average. In fact, the host ranges of the majority of studied phages in this collection span multiple hosts in the clinical Kp community, with an average of 19.59 in group 2 and 21.27 in group 3; however, the majority of phages from these groups exhibit lower infectivity to the hosts. In parallel, strains were susceptible to multiple phages (mean = 30.66 ± 18.33). The above findings appear to expand the knowledge of phage specialization, highlighting the flexibility in phage host specificity.

### Engineering efficient phage-antibiotic synergy combinations

**Fig 3:**
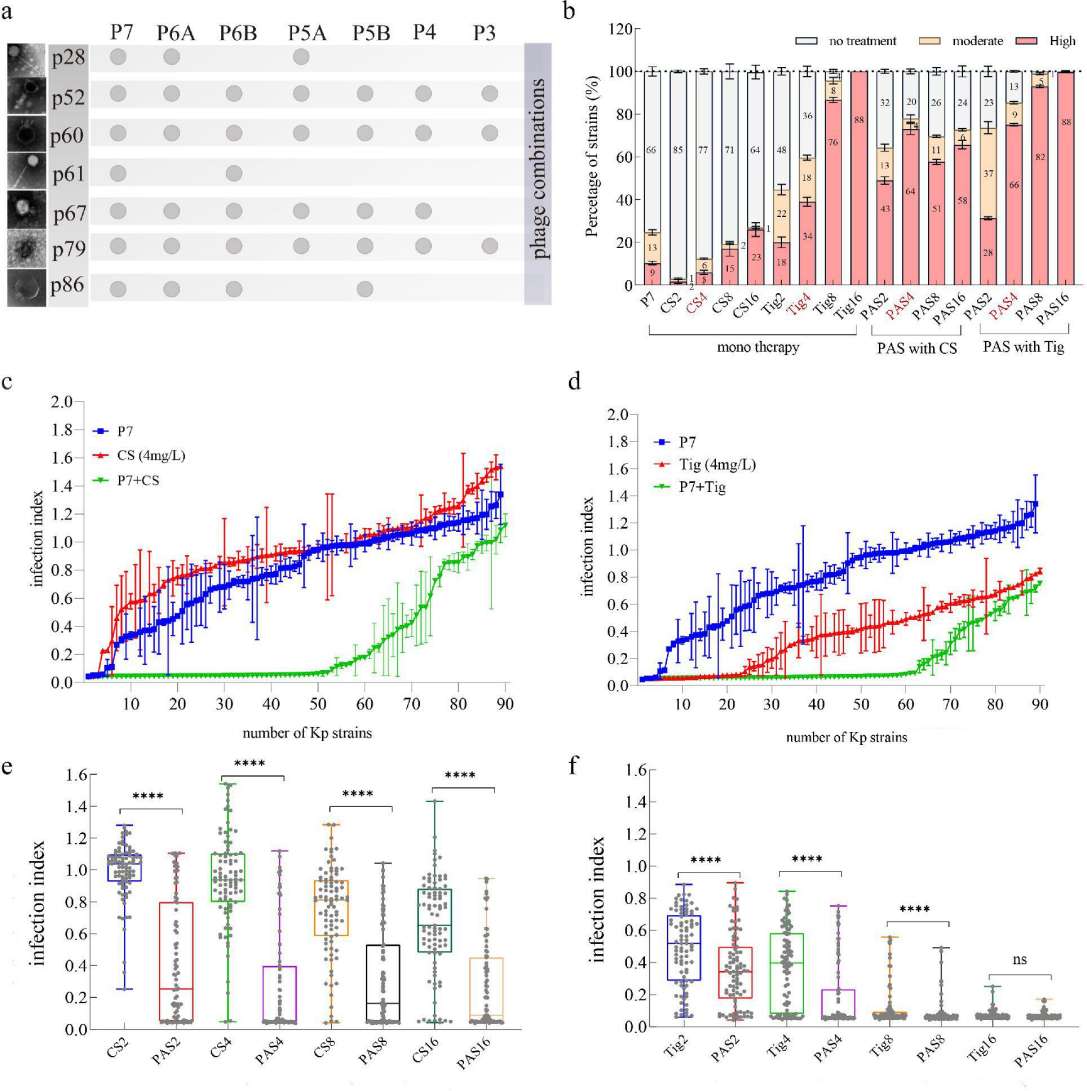
The design of phage cocktail combinations and comparisons of bactericidal effectiveness among different treatment approaches. (**a**) The negative staining electron microscopy image of seven phages (left) and the phage combinations for phage cocktails (right). “*P*” denotes phage cocktails with the subsequent number indicating the number of phages used in the phage cocktails, for example, P3 indicates a cocktail composed of three different phages. (**b**) The proportion of no, moderate, and high bactericidal effectiveness against 88 clinical MDR Kp strains by phage cocktail (**P7**) in combination with colistin or tigecycline. The threshold values of no (index >0.5), moderate (0.2 < index ≤ .5), and high bactericidal activity (index ≤0.2) were determined as the mean ± SEM of ratios of bacterial density between treated and non-treated groups (OD_600-treated_/OD_600-non-treated_), respectively. The strain numbers were included in each bar chart. (**c-**d) The comparison of antibacterial activity among phage-only (**P7**), antibiotic-only, and PAS treatments, indicating that PAS treatment suppressed a higher number of MDR Kp strains. The plotted values represent mean ± SEM (*n* = 3). (**e-f**) The average values of infection index for seven-phage cocktails (**P7**) with or without antibiotics, against 88 clinical MDR Kp strains. Three independent replicates were performed for each treatment. CS2/4/8/16 indicates the mono-antibiotic therapy with 2, 4, 8, or 16 mg/L colistin, respectively; Tig2/4/8/16 indicates the mono-antibiotic therapy with 2, 4, 8, or 16 mg/L tigecycline, respectively. The abbreviations used for each treatment are defined as follows: P7 denotes phage cocktails containing seven phages (refer to main Fig. 3A); PAS represents P7 combined with antibiotics with subsequent numbers indicating the antibiotics concentrations. For instance, PAS2 represents P7 combined with 2 mg/L colistin (CS) or tigecycline (Tig).

To improve the possible outcome of phage therapy for bacterial suppression, we designed the best possible phage cocktail combinations through PhageCocktail R package, based on phage lytic activity and host ranges. A total of seven phages were selected, which were distributed in different groups (Fig. 2), and the negative staining electron microscopy images indicate that seven selected phages possess three different morphological phages (Fig. 3a; Fig. S1), phage P52 with a contractile tail, four phages with a short tail (P60, P67, P79, and P85), and two phages containing a long non-contractile tail (P28 and P61). The whole genome sequences revealed that these seven phages belong to *Caudoviricetes* class (P85 and P61), *Slopekvirus* genus (P52 and P60), *Webervirus* genus (P28), and *Przondovirus* genus (P67 and P79). Phage genomes did not carry antibiotic resistance or virulent genes when screened against the relevant databases (Fig. S2 to 8). The circular genetic characteristics of these seven phages are available in Fig. S2 to S8.

These seven phage cocktails were tested by challenging them against 88 MDR Kp strains individually. The infection index was used to evaluate the efficacy of phage treatments in reducing bacterial density, which involves determining the ratio of bacterial density in the treated (e.g., antibiotic-treated, phage-treated, or combination-treated) to that in the non-treated control group. The reduction in the incidence of disease could be explained by a reduction in pathogen densities, with a profound effect observed as the number of phages in each cocktail increased. Notably, the seven-phage combination (P7) displayed the highest therapeutic efficacy, significantly reducing the infection index in a greater proportion of strains compared with other phage cocktails (10.33% with infection index <0.2, Fig. S9). Because the outcomes of two variants of five-phage cocktails (P5A and P5B) and six-phage cocktails (P6A and P6B) were similar, we only selected P5B and P6B for later analysis. Similar to antibiotic treatments, monotherapy of phage cocktails only targeted a subset of tested strains, for instance, P7 was only able to effectively suppress 10 out of 88 MDR Kp strains.

To achieve a broader therapy efficacy, we sought to develop PAS combinations by combining phage cocktails with two last-resort antibiotics (colistin and tigecycline,). The combination of antibiotics with an increasing number of phage cocktails resulted in a noticeable reduction in bacterial density (Fig. 3b; Fig. S10 and 11**)**. Specifically, phage cocktails combined with colistin increased the number of effectively suppressed MDR Kp strains (infection index <0.2) from 5 (4 mg/L CS only) to 64 (P7+CS4) out of 88 MDR Kp strains (Fig.3b). Similarly, approximately 34 and 66 MDR Kp strains were eliminated by the Tig4 only and P7+Tig4 combinations, respectively (Fig.3b). This synergistic effect of PAS with either colistin or tigecycline can be observed in all phage cocktails, even with the lowest number of phage cocktails (P3) (Fig. S10 and S11), resulting in a greater reduction in pathogen density compared with phage monotherapy (Fig. 3c and d). Notably, colistin, a cell membrane disrupter, and tigecycline, an inhibitor of bacterial protein translation, exhibited different interactions with the same phage cocktails (Fig. 3e and f; Fig. S10-S11). These results indicate PAS can synergistically enhance bactericidal effectiveness.

### Assessment of phage-antibiotic efficacy in an *in vivo* mice model

**Fig 4:**
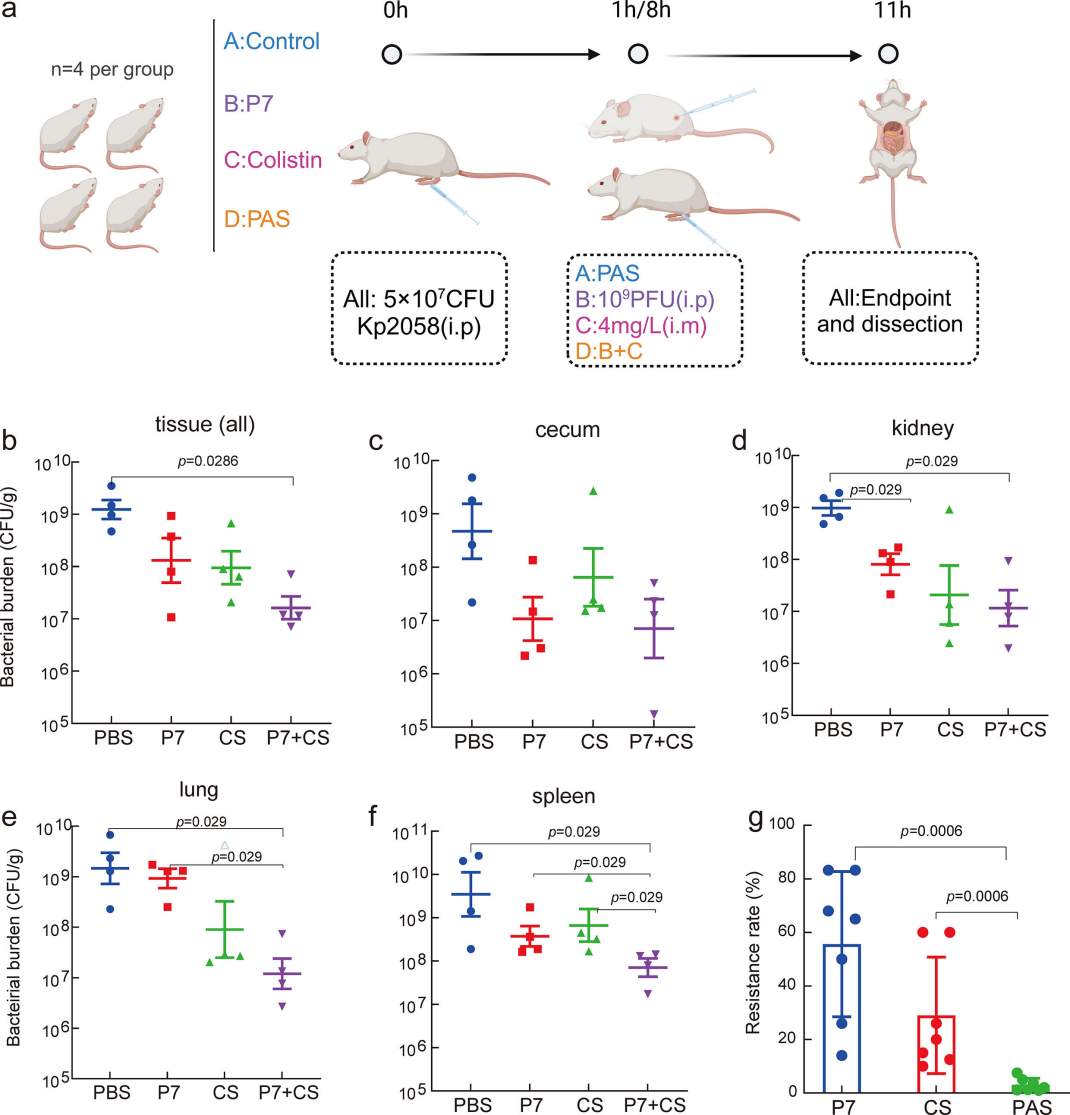
*In vivo* assessment of phage-antibiotic combination therapy against *K. pneumoniae* infection. (a) an overview of the *in vivo* experiment using murine model, created with Biorender. (**b-**f) Bacterial burden of *K. pneumoniae* in different tissues at endpoint 11 h, comparing treatments: control (blue), phage only (red), colistin only (green), and combined therapy (purple). Each data point represents one mouse with bars indicating mean and SEM (*n* = 4). (g) Bacterial resistance rates corresponding to different treatments. Seven colonies were isolated from each treatment group, and resistance rates were determined by a standard agar overlay assay. Statistical analysis was performed using Mann-Whitney unpaired *t*-test.

We evaluated the efficacy of PAS in a murine model using Kp2058 strain, belonging to ST11 CRKp group with hypervirulent genes. Mice (*n* = 4 per group) were treated with PBS) (negative control), colistin alone, phage cocktail P7, or a combination of colistin and P7 (Fig. 4a). Compared with non-treatment group (control), the PAS treatment demonstrated superior efficacy, reducing bacterial density by 2.01 log_10_ units (Fig. 4b, Mann-Whitney *t*-test, *P* = 0.0286). Specifically, bacterial burden reductions were 0.84 log_10_ units with P7 alone and 1.19 log_10_ units with colistin alone. Similar trends were observed in individual organs (cecum, kidney, lung, and spleen, Fig. 4c through f). For example, PAS treatment reduced bacterial density by 1.72, 0.41, and 0.57 log_10_ units, against the control, colistin-only, and P7-only groups, respectively (Fig. 4e, Mann-Whitney *t*-test, all *P* = 0.0286).

We further evaluated bacterial resistance to phage and PAS treatments by randomly selecting seven bacterial clones from each treatment group. The bacterial resistance rates were estimated by the ratio of the number of resistance colonies to total bacterial cells. Strains recovered from P7 treatment group exhibited the highest resistance rate (mean = 55.66%), followed by colistin group (mean = 29.07%). In contrast, bacteria from the PAS group showed the lowest resistance rate (mean = 3.75%), significantly lower than both monotherapy groups (Fig. 4g, Mann-Whitney *t*-test, *P* = 0.0003–0.0086). Taken together, our *in vivo* findings demonstrate that the PAS therapy is more effective than monotherapy with either phage or colistin against *K. pneumoniae* infection. PAS therapy significantly inhibits the colonization of pathogenic bacteria and reduces the emergence of phage and antibiotic resistance.

## DISCUSSION

Since the alarmingly rapid dissemination of MDR bacterial pathogens, such as *K. pneumoniae*, alternative therapeutic strategies have become imperative (22, 23). Our results feature several potentially important implications. First, we have demonstrated superior therapeutic outcomes of phage-antibiotic synergetic therapy targeting MDR *K. pneumoniae* pathogens, CRKp in particular. During optimization experiments, we observed that the number of phage cocktails and the dose of antibiotics have significant effects on antibacterial activity and the development of resistance (Fig. 3 and 4). Previous *in vitro* findings illustrated the efficacy of *Klebsiella-*specific phages (11–13, 24–26) or phage-antibiotic combinations (14) against a single *K. pneumoniae* strain. However, there is a pressing need for broader spectrum antibiotic-phage combinations capable of suppressing different strains of this pathogen. In this study, PAS therapy combining phage cocktails with colistin or tigecycline demonstrated potent antibacterial activity against the majority of 88 clinical MDR *K. pneumoniae* strains (*n* = 64%-66%, 73%-75%, Fig. 3). Importantly, the emergence of resistance to PAS was significantly decreased from 55.66% to 3.75% (Fig. 4g), suggesting its potential as an effective therapeutic option.

The effectiveness of PAS therapies can be attributed to the distinct antibacterial mechanisms of lytic phages in complement with antibiotic actions, alongside their ability to replicate selectively in response to bacterial populations. Recent reviews have discussed the potential advantages of using PAS as antibacterial therapeutics against MDR infections (27–29), including *Staphylococcus aureus* (19), *Pseudomonas aeruginosa* (30), *K. pneumoniae* (14), and *Acinetobacter baumannii* (10). However, most of those studies, if not all, focused on developing phages targeting specific pathogenic strains, reflecting a personalized therapeutic approach (31). Our study expands on this concept by demonstrating the efficacy of optimized PAS therapy across multiple clades of clinical MDR Kp strains (*n* = 66/88, 75%). Furthermore, our *in vivo* assessment of PAS efficacy against one representative ST11 CRKp strain provides the cornerstone for anti-infective drug development and could be further evaluated by more preclinical studies.

There is a never-ending “arms race” between bacteria and their phages, wherein bacteria have evolved acquiring numerous defense systems to resist phage infection, and phages have reciprocally developed counter-defense strategies through Darwinian selection (32–35). Consequently, phage resistance poses a considerable threat to treatment efficacy. Current strategies often involve the use of multi-phage cocktails (36) or preadapted phages (37) to combat resistance. For example, Borin et al. (38) found that λ phages evolved through serial passage for 28 days exhibited enhanced bacterial suppression and delayed resistance emergence. Despite promising results, the ongoing evolution between bacteria and phages makes the success of phage training uncertain (32, 39, 40). Another approach is to use phage cocktails consisting of multiple phages to broaden antimicrobial activity spectrum and mitigate phage resistance development. It is rational to hypothesize that bacteria face greater difficulty developing resistance to phages with multiple receptors, thereby resulting resistance likelihood (36, 41, 42). Importantly, phage-resistant bacteria often confer a fitness burden resulting in resensitizing to antibiotics (43). Hence, phage-antibiotic combinations may enhance bacterial growth suppression without promoting resistance emergence.

In conclusion, our study provides promising evidence that optimized PAS therapy has considerable potential for suppressing multiple clades of clinical MDR *K. pneumoniae* pathogens. To optimize phage therapy efficacy against MDR *K. pneumoniae* strains, we propose three strategies: (i) selection of broad host range phages targeting diverse MDR *K. pneumoniae* strains, given the profound implications of phage host range on interactions and broad-spectrum protection against pathogenic bacteria; (ii) Formulation optimization of phage-based on phage lytic capacity and host range specificity, maximizing their implications against diverse bacterial targets; and (iii) combinations with last-line antibiotics that remain effective to MDR *K. pneumoniae* strains. Such optimized PAS treatments enhance their capacity for bacterial suppression and delay the evolution of phage resistance in both *in vitro* and *in vivo* models. Furthermore, PAS combination could be advantageous for a better understanding of bacterial pathogenesis and genetic mechanisms governing sensitivity and resistance to phage-antibiotic combinations.

## MATERIALS AND METHODS

### Strains and bacterial whole genome sequencing

A total of 88 clinical MDR *K. pneumoniae* strains obtained from patients from 2018 to 2020 were selected in this study (Data S1). These strains were routinely grown at 37°C on LB agar plates or in LB broth medium for 20 h with shaking (180 r.p.m) before all the experiments. Bacterial genome sequencing was conducted by the Illumina platform. Bacterial DNA was extracted using genomic DNA (gDNA) extraction kit (TIANGEN, China) following the manufacturer’s protocol. The concentration of each gDNA sample was determined using a Qubit Flex System (Invitrogen, United States). Raw Illumina reads were filtered with Trimmomatic v. 0.38.1 (44) with default parameters. Sequences were assembled by SPAdes and annotated using Prokka v. 1.14.6 (Galaxy version) (45). The genetic characteristics of 88 MDR Kp strains, including bacterial taxonomy, MLST, and resistance genes, were further analyzed against the ResFinder, plasmidFinder, and pointFinder databases using the staramer tool (Galaxy version 0.9.1). Phylogenic trees were constructed by snippy (v4.4.4) based on bacterial core genome alignment. All sequencing data are available in the NCBI sequence Read Archive, with BioProject accession no: PRJNA985089 and BioSample accession No. from SAMN35788486 to SAMN35788567.

### Enrichment and isolation of phages from wastewater

Wastewater samples were collected from three Wastewater Treatment Plants in Fuzhou, China. Ten microliters of sewage samples were centrifugated at 12,000 rpm for 3 min at 28°C and filtered using 0.22 µm microporous membrane three times. Before phage isolation, 88 clinical MDR *K. pneumoniae* strains (defined as Kp strains in the following text) were randomly divided into 10 groups of strain cocktails. A total of 1 mL phage filtrate was added and fully mixed with 10 groups of strain cocktails, respectively, followed by co-incubation for 24 h at 37℃. Then, isolate and purify phages separately from each strain of *K. pneumoniae.* The double-layer plaque assay was performed to determine the infectivity of the enriched bacteriophages against 88 MDR Kp strains. The agar plates contain two layers of agar using 1.5% LB as the bottom agar and 0.6% soft LB agar as the top agar containing phage and bacteria mixture. A single transparent plaque was transferred to 2 mL fresh LB broth containing 500 µL of bacteria host and co-incubated at 37℃ for 16 h. The phage was purified at least three times until a single morphology of the plaque was observed.

### Determination of phage host ranges

Phage host ranges were assayed on 88 clinical MDR Kp strains using spot lysis assay as previously described (46). In brief, 5 µL of each phage lysate (*n* = 100) was spotted in duplicates onto an agar plate containing one MDR Kp strain and incubated for 16 h at 37°C. If single lysis plaques appeared after overnight incubation, the phage was considered infectable to the respective host, and the lytic host range was reported. The host range spectrum of the phages was further analyzed according to morphology of the spot plaque formed, which can be classified into five groups: (i) complete clearing (defined value of 1.00); (ii) clearing throughout but with faintly hazy background (defined value of 0.75); (iii) substantial turbidity throughout the cleared zone (defined value of 0.75); (iv) a few individual plaques (defined value of 0.25); and (v) no plaques (defined value of 0.00).

### Transmission electron microscopy

The phages were propagated with host bacteria at 37°C for overnight incubation. The single lytic plaque was picked into 500 µL PBS buffer and mixed well. A total of 20 µL of phage suspension was added to the carbon-coated copper grids for 5 min, and the grids were washed in sterile water and dried with filter paper. The absorbed phage particles were negatively stained for 10 min with 1% phosphotungstate acid. The phage morphology was observed on a Hitachi HT7700 microscope.

### Phage DNA extraction and whole genome sequence

The single lytic plaque was eluted in 5 mL of SM buffer to the plate and centrifuged at 12,000 rpm for 3 min to remove residual bacteria. The phage suspension was repeatedly filtered with a 0.22 µm syringe filter for three times. The purified phage DNA was extracted with a viral DNA extraction kit (Blood & Tissue kit, QIAGEN), in accordance with the manufacturer’s guidelines. The DNA concentration was measured using a Qubit Flex fluorometer (Thermo Fisher, USA) and subjected to whole genome sequence using the Novaseq platform. Raw reads were trimmed using the trimming tool of CLC Genomics Workbench software (version 11.0.1). The contaminated bacterial host genomic sequences were removed, and the resulting clean phage sequences were assembled using SPAdes method. Open reading frames were annotated using Prokka with blastn against the NCBI database. The presence of ARGs, virulence, MGEs, and integrases in phage genomes was screened against relevant databases, including staramr (Galaxy version 0.9.1) and ABRicate (Galaxy version 1.0.1). The whole genome sequences of seven isolated phages for cocktail combinations were submitted to NCBI via BankIt with Genbank accession No. OR256020 (P28), OR256021 (P52), OR256022 (P60), OR256024 (P61), OR256025 (P85), OR256026 (P67), and OR256027 (P79).

### Determination of bactericidal efficiency of phage-monotherapy, antibiotics-monotherapy, and PAS therapy

Based on phage-bacterial infection networks (phage host ranges data), we designed the best possible phage cocktail combinations through the R package called PhageCocktail using the ExhaustiveSearch method (47). To determine the bactericidal efficacy of monotherapy (antibiotic- or phage-only) and combined treatments against 88 MDR Kp strains, bactericidal kinetic experiments were performed. In brief, 88 Kp strains were inoculated in 96-well plates overnight at 37°C with 180 r.p.m, the next day, the overnight culture of the tested strains was then approx.1:200 diluted to bacterial OD_600_ 0.1–0.15. For the preparation of phage cocktails, each single phage was diluted to 108 PFU/mL and then 1:1 mixed with other phages. Four different treatments were challenged against bacterial cultures: (i) the mono-treatment of phage cocktails (with approximately 10^9^ PFU/mL per phage); (ii) the mono-treatment of antibiotic, with the different concentrations of colistin or tigecycline; (iii) the combination of phage cocktail and antibiotics (colistin or tigecycline); and (iv) a negative control group without any treatment. Three biological replicates were performed in each group, and bacterial densities were measured as the optical density at 600 nm (OD_600_) every hour by SpectraMax iD3 (Molecular Devices, United States) for 24 h at 37°C. The bacterial infection index (at 24 h) can be used to estimate the potential bactericidal efficacy of different treatments, by comparing with the negative control group: infection index = [OD_600treated_/OD_600non-treated_], which is similar to a previous study (48). The infection index was normalized to a figure between 0 to 1: infection index ≤0.2, high bactericidal activity; 0.2 < index ≤ .5, moderate bactericidal activity; and index >0.5, little or no bactericidal activity. Three independent repeats were performed for each treatment.

### *In vivo* murine model of CRKp infection

The specific-pathogen-free (SPF) BALB/c mice, male, 5–7 weeks old, weighing at least 18 g, were used for *in vivo* murine infection models. In order to build a *K. pneumoniae* infection model, mice were intraperitoneally injected with 80 mg/kg cyclophosphamide for 3 days before intraperitoneally infected with a CRKp strain 2058 (5 × 10^7^ CFU/mL), a representative of a high-risk clone of ST11 CRKp strains in this study. The infected mice were randomly divided into four experimental groups (*n* = 4 per group) and after bacterial infection at 1 h and 8 h, mice were, respectively, received either (i) PBS(control); (ii) 4 mg/kg colistin; (iii) 200 µL of phage cocktail P7 (10^8^ PFU/mL); or (iv) the combination of colistin (4 mg/kg) and phage cocktail P7 (200 µL, 10^8^ PFU/mL). To quantify phage and bacterial burden in tissues, mice were euthanized at 11 h after bacterial infection. The kidney, spleen, lung, and cecum of each mouse were then collected, weighed, and homogenized in 500 µL of PBS. Bacterial loads and phage plaque forming unit (PFU) quantification were quantified by colony forming unit (CFU) counting and double-agar-layer method, respectively.

To estimate the bacterial resistance rate to different treatments, seven colonies per treatment were recovered from different tissues of mice. Overnight culture of each selected bacteria was prepared and subsequently diluted into sterile saline (up to 10^−6^ dilutions). An aliquot of 10 µL of the culture was plated on different selective agar medium and incubated for 24 h at 37°C. For instance, colonies isolated from the P7 group were plated on LB agar plates supplemented with the P7 cocktail. Bacterial densities were quantified by CFU, and the bacterial resistance rate in the mice was calculated using the formula:


$$CFU_{selective\;plate}/CFU_{non\;selective\;plate}\;\times \;100.$$

### Statistical analysis

Data analysis was performed using GraphPad Prism (8.3.1). Data shown in plots are represented as the mean of at least two replicates ± SEM, and an exact number of independent replicates for each experiment is stated in their respective figure legends. Mann-Whitney *t*-test analysis (*P* < 0.05) was used to compare differences in bacterial growth rates and fitness assay.

## Data Availability

The data that support the findings of this study are provided within the manuscript and its associated supplementary appendix. All whole genome sequences of 88 MDR Klebsiella pneumoniae strains were submitted to the national center for biotechnology information (NCBI) number with BioProject ID PRJNA985089 (BioSample: from SAMN35788486 to SAMN35788567), the whole genome sequences of seven isolated phages for cocktail combinations were submitted to NCBI via BankIt with Genbank accession No. OR256020 (P28), OR256021 (P52), OR256022 (P60), OR256024 (P61), OR256025 (P85), OR256026 (P67), and OR256027 (P79).

## SUPPLEMENTAL MATERIAL

Data Set S1 msystems.00607-24-s0001.xlsx Strain information and source data for Fig. 2.

Supplemental figures msystems.00607-24-s0002.pdf Fig. S1 to S11.

ASM does not own the copyrights to Supplemental Material that may be linked to, or accessed through, an article. The authors have granted ASM a non-exclusive, world-wide license to publish the Supplemental Material files. Please contact the corresponding author directly for reuse.
