# Supplementary material for: Antibacterial effect of phage cocktails and phage-antibiotic synergy against pathogenic Klebsiella pneumoniae
Source: mSystems. 2024 Aug 21;9(9):e00607-24. doi: 10.1128/msystems.00607-24 (PMC11406915; doi:10.1128/msystems.00607-24)
Supplement: Supplemental figures — Fig. S1 to S11. [file msystems.00607-24-s0002.pdf]

## Supplementary files for

# Antibacterial effect of phage cocktails and phage-antibiotic synergy against pathogenic *Klebsiella pneumoniae*

Mengshi Zhao[1,2#], Hongru Li[3#], Dehao Gan[2], Mengzhu Wang[1,2], Hui Deng[2*], Qiu E. Yang[1*]

1. College of Resources and Environment, Fujian Agriculture and Forestry University, Fuzhou 350002, China.
2. Fujian Key Laboratory of Traditional Chinese Veterinary Medicine and Animal Health, Fujian Agriculture and Forestry University, Fuzhou 350002, China
3. Department of Pulmonary and Critical Care Medicine, Fujian Shengli Medical College, Fujian Medical University, Fujian Provincial Hospital, Fuzhou 350001, China

[#] These authors contributed equally to this work.

*For Correspondence: Prof. Qiu E. Yang (qiueyang2014@163.com) and Dr. Hui Deng (deng12345hui@163.com).

**Supplementary Figure S1-11**

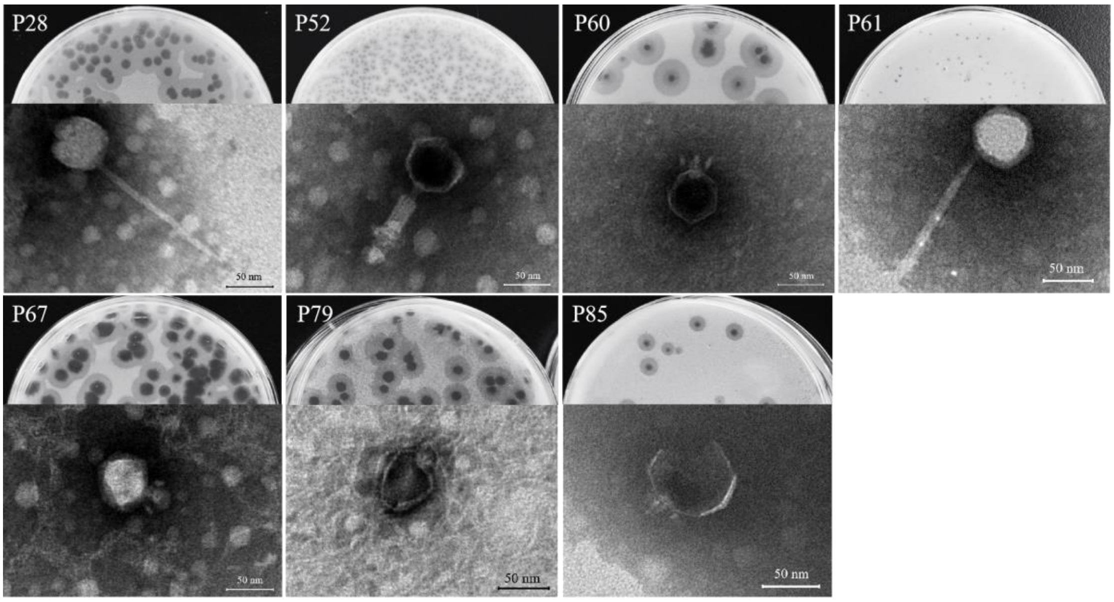


**Fig.S1** Plaque morphologies and representative microscopic images of seven phages selected for phage cocktail combination. Clear plaque morphologies depict the distinct shapes and sizes formed by each phage on bacterial lawns, indicative of their lytic activity and propagation characteristics. Their representative microscopic images provide a detailed visualization of the morphology of each phage, showing their typical capsid structure and tail morphology. The scale bar for TEM images was 50 nm.

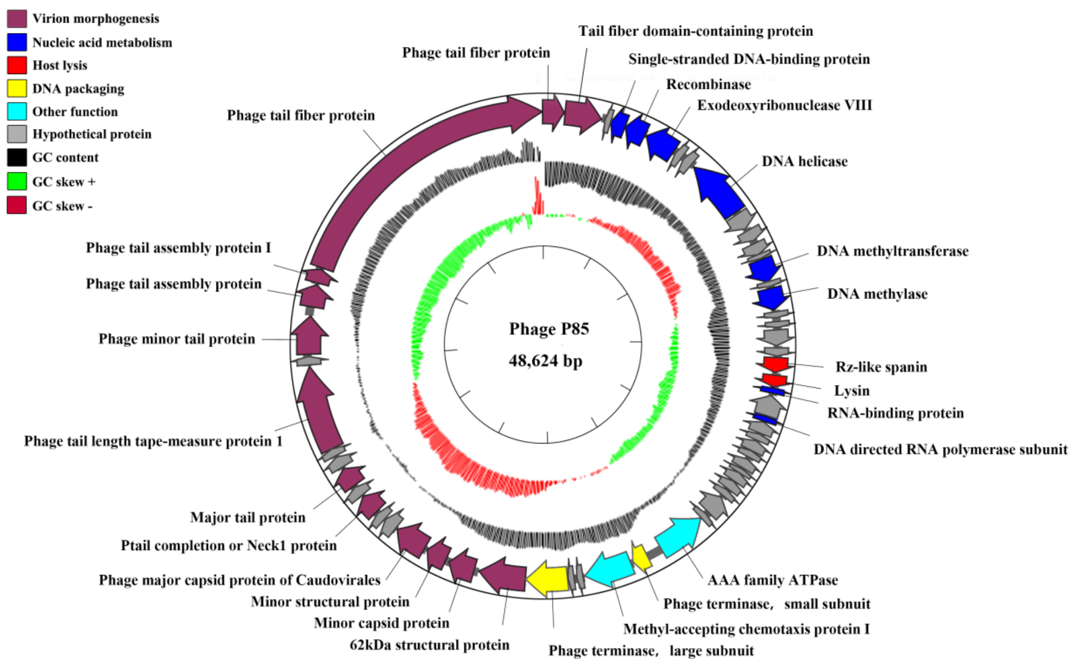


**Fig.S2 The circular genomic map of phage P85 with annotated features.** The color-coded functional categories are indicated as follow: virion morphogenesis (purple), nucleic acid metabolism (blue), host lysis (red), DNA packing (yellow) and other functions (wathet). The first two inner circles show the GC skew (+ green, -crimson) and GC content (black), respectively. Proteins with unknown functions are marked with gray. The genome size of the phage is indicated in the inner most circle.

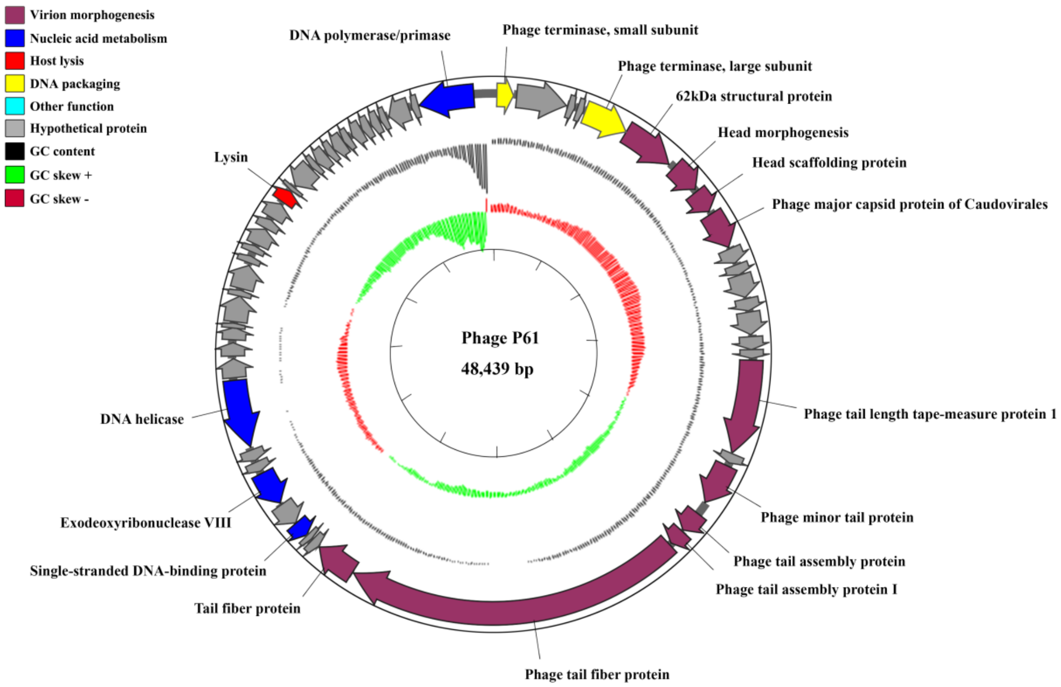


**Fig.S3 The circular genomic map of phage P61 with annotated features.** The color-coded functional categories are indicated as follow: virion morphogenesis (purple), nucleic acid metabolism (blue), host lysis (red), DNA packing (yellow) and other functions (wathet). The first two inner circles show the GC skew (+ green, -crimson) and GC content (black), respectively. Proteins with unknown functions are marked with gray. The genome size of the phage is indicated in the inner most circle.

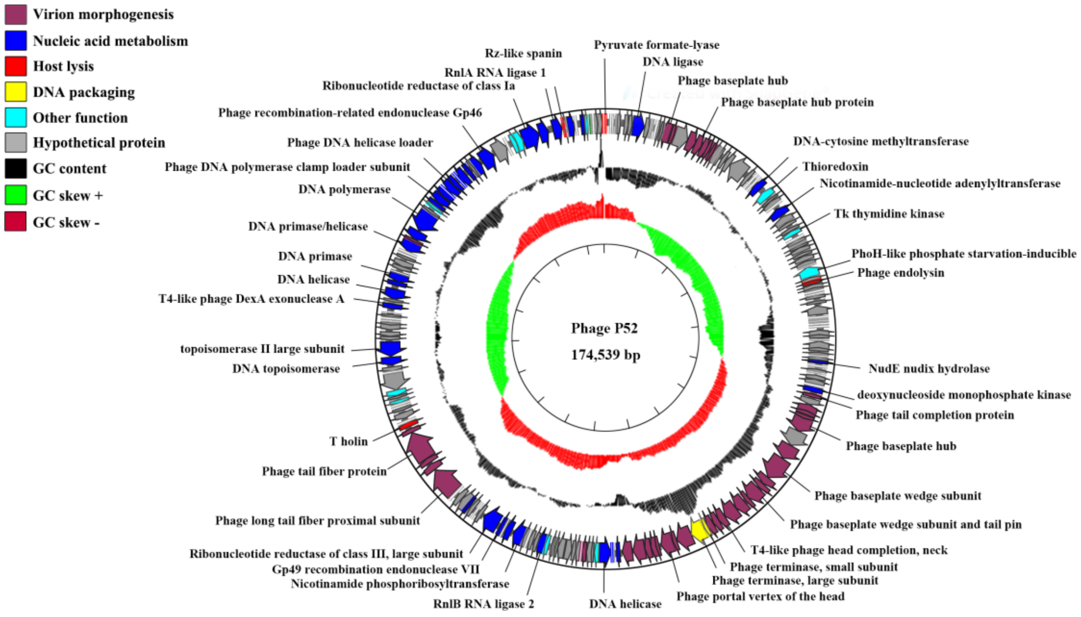


**Fig.S4 The circular genomic map of phage P52 with annotated features.** The color-coded functional categories are indicated as follow: virion morphogenesis (purple), nucleic acid metabolism (blue), host lysis (red), DNA packing (yellow) and other functions (wathet). The first two inner circles show the GC skew (+ green, -crimson) and GC content (black), respectively. Proteins with unknown functions are marked with gray. The genome size of the phage is indicated in the inner most circle.

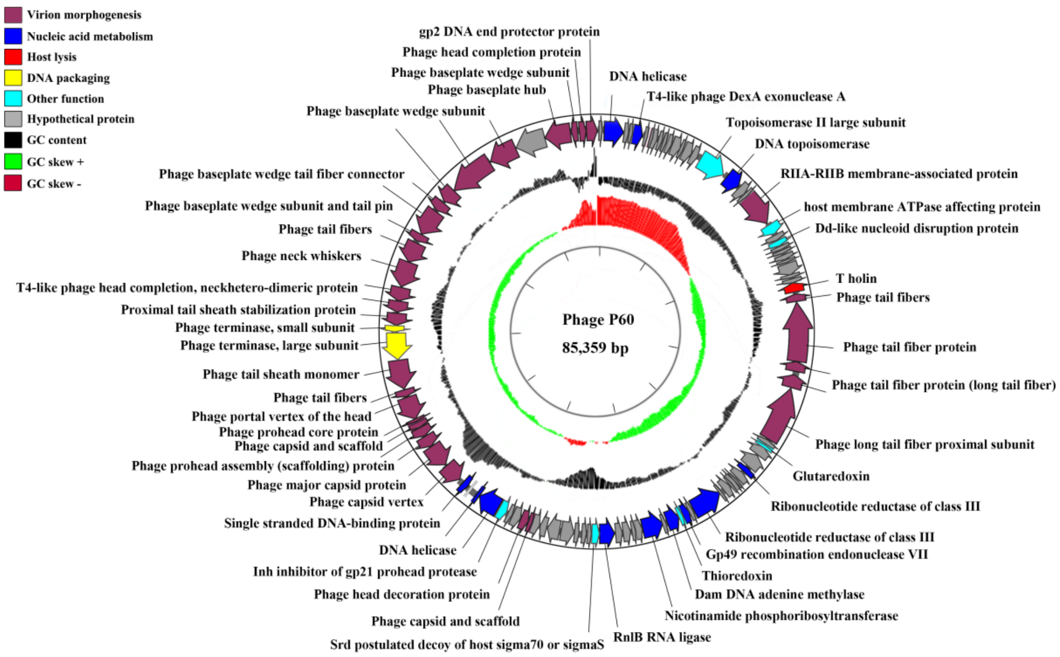


**Fig.S5 The circular genomic map of phage P60 with annotated features.** The color-coded functional categories are indicated as follow: virion morphogenesis (purple), nucleic acid metabolism (blue), host lysis (red), DNA packing (yellow) and other functions (wathet). The first two inner circles show the GC skew (+ green, -crimson) and GC content (black), respectively. Proteins with unknown functions are marked with gray. The genome size of the phage is indicated in the inner most circle.

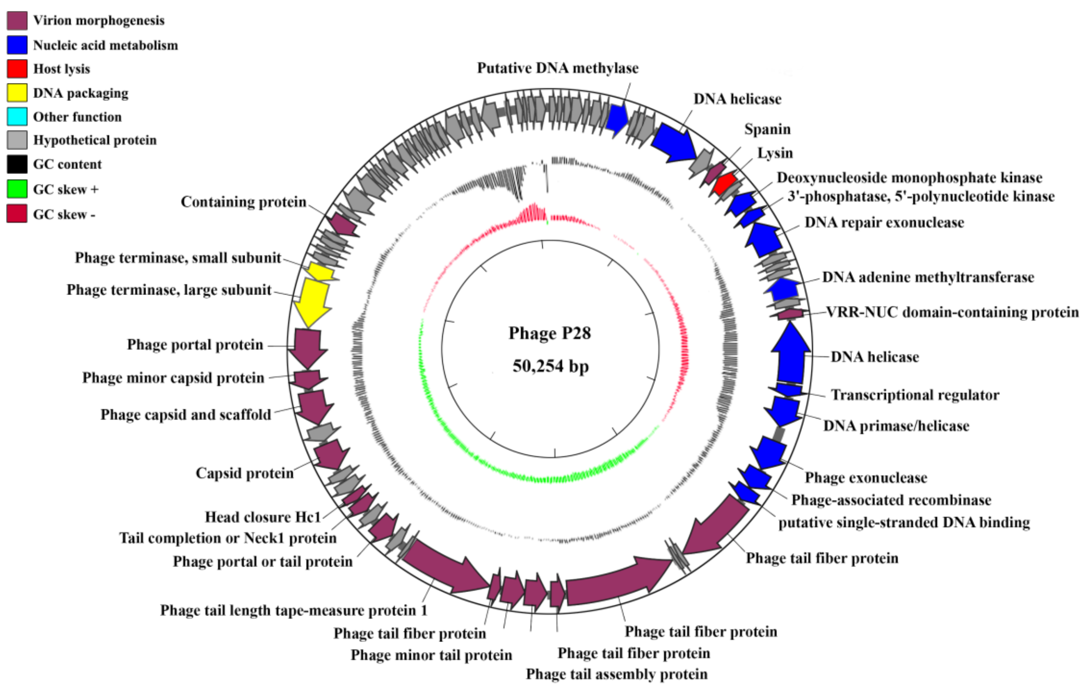


**Fig.S6 The circular genomic map of phage P28 with annotated features.** The color-coded functional categories are indicated as follow: virion morphogenesis (purple), nucleic acid metabolism (blue), host lysis (red), DNA packing (yellow) and other functions (wathet). The first two inner circles show the GC skew (+ green, -crimson) and GC content (black), respectively. Proteins with unknown functions are marked with gray. The genome size of the phage is indicated in the inner most circle.

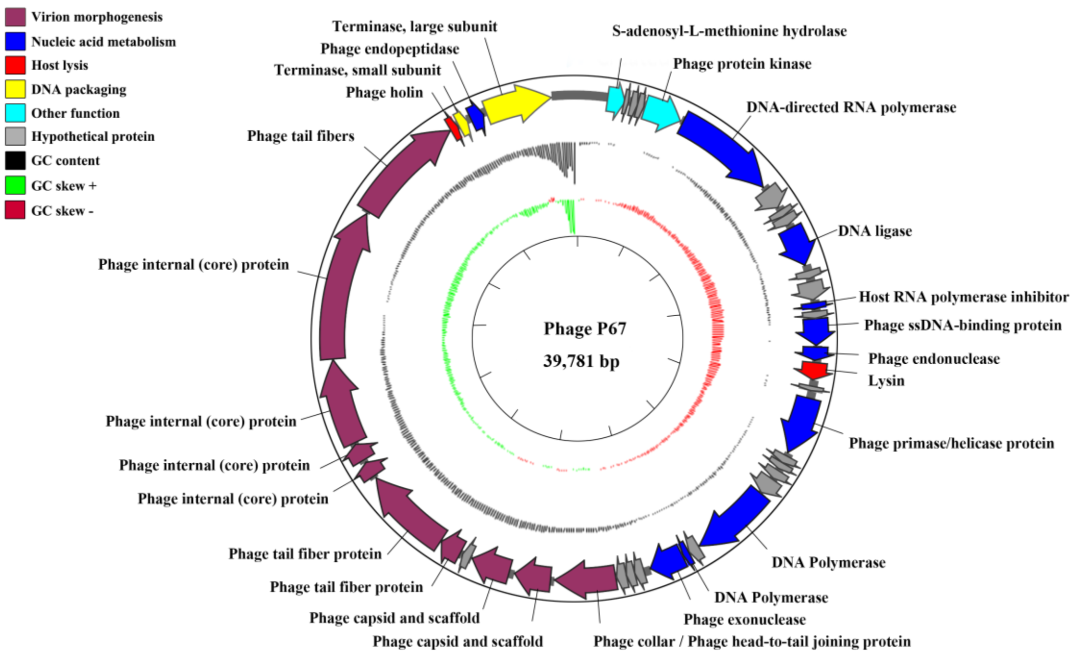


**Fig.S7 The circular genomic map of phage P67 with annotated features.** The color-coded functional categories are indicated as follow: virion morphogenesis (purple), nucleic acid metabolism (blue), host lysis (red), DNA packing (yellow) and other functions (wathet). The first two inner circles show the GC skew (+ green, -crimson) and GC content (black), respectively. Proteins with unknown functions are marked with gray. The genome size of the phage is indicated in the inner most circle.

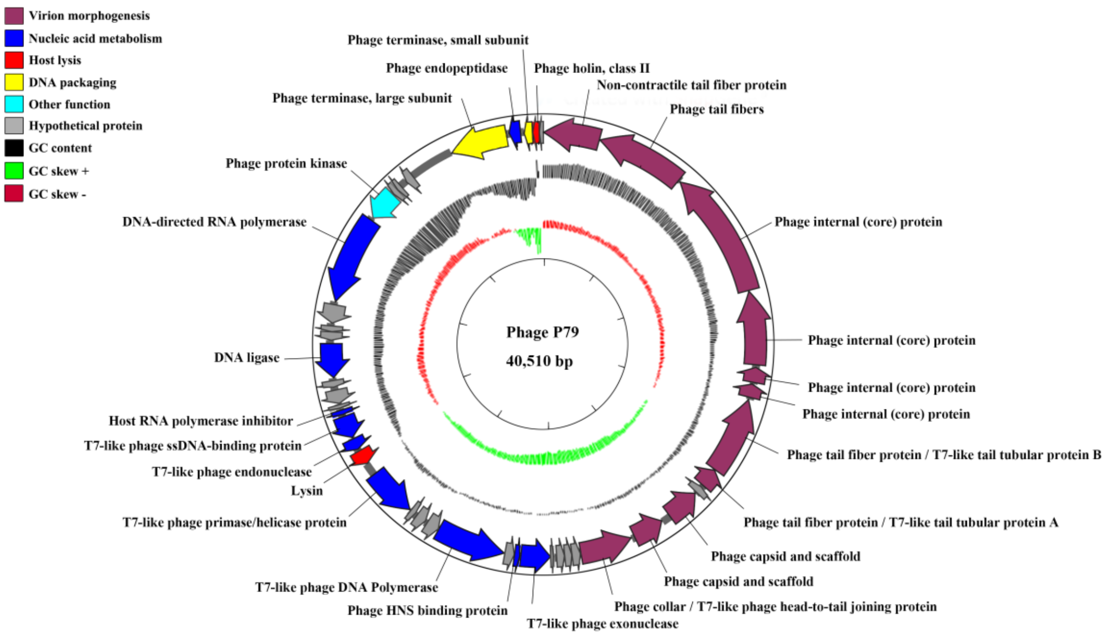


**Fig.S8 The circular genomic map of phage P79 with annotated features.** The color-coded functional categories are indicated as follow: virion morphogenesis (purple), nucleic acid metabolism (blue), host lysis (red), DNA packing (yellow) and other functions (wathet). The first two inner circles show the GC skew (+ green, -crimson) and GC content (black), respectively. Proteins with unknown functions are marked with gray. The genome size of the phage is indicated in the inner most circle.

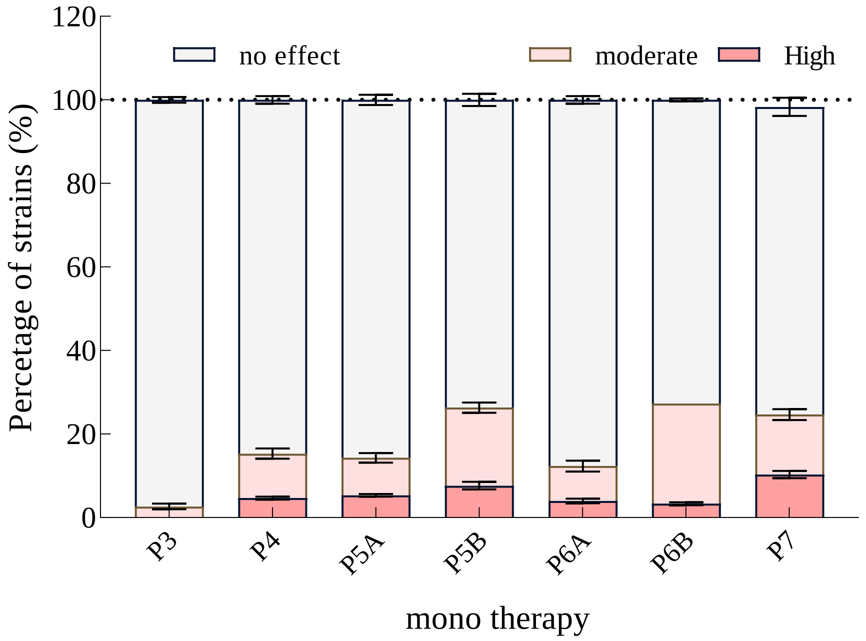


**Fig.S9 The proportion of no, moderate and high bactericidal effectiveness against 88 clinical MDR Kp strains by different phage cocktails.** The threshold values of no (index>0.5), moderate (0.2<index≤0.5) and high bactericidal activity (index≤0.2), were determined as the ratios of bacterial density between treated and non-treated group ($OD_{600\text{-treated}}/OD_{600\text{-non-treated}}$), respectively. Three independent replicates were performed for each treatment and the abbreviations for phage cocktail combinations were indicated in main Fig.3a. "P" denotes phage cocktails with the subsequent number indicating the number of phages used in the phage cocktails, for example, P3 indicates a cocktail composed of three different phages.

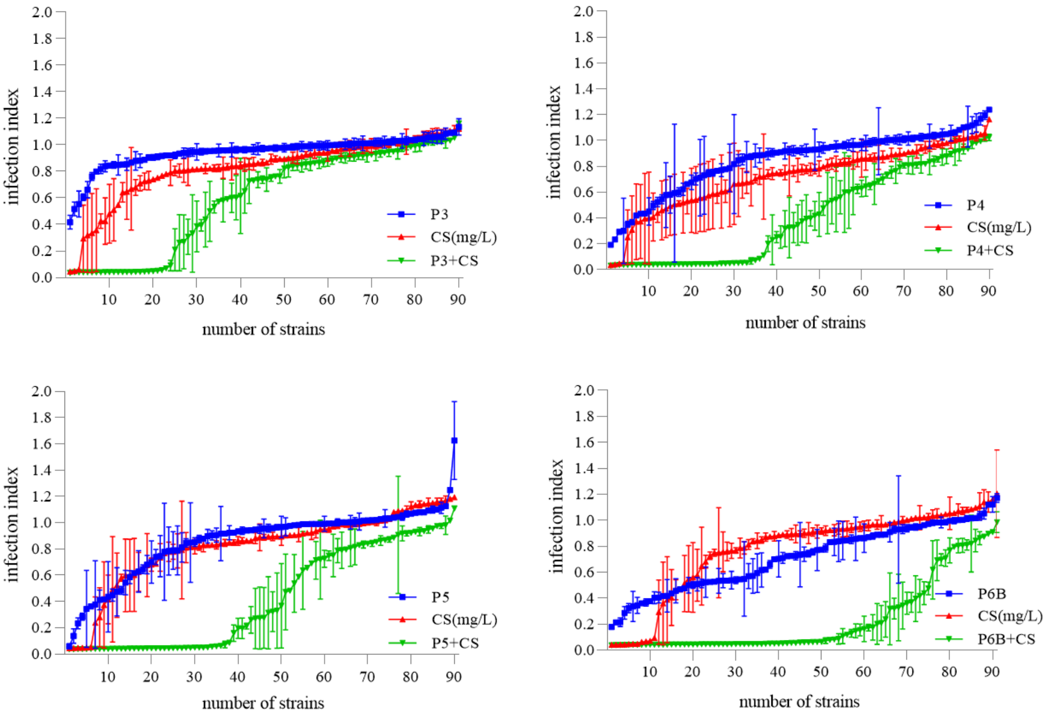


**Fig.S10 *In vitro* antimicrobial activity of four phage cocktails (P3-P6) with or without colistin (4 mg/L) against 88 MDR Kp strains.** The infection index was indicated as the ratio of bacterial density between treated and non-treated groups ($OD_{600\text{-treated}}/OD_{600\text{-non-treated}}$). The threshold value for high bactericidal activity was 0.2, which indicate that the phage-cocktail combination effectively suppressed a higher number of MDR Kp strains. “P” denotes phage cocktails with the subsequent number indicating the number of phages used in the phage cocktails, for example, P3 indicates a cocktail composed of three different phages. Three independent replicates were performed for each treatment and the abbreviations for phage cocktail combinations were indicated in main Fig.3a.

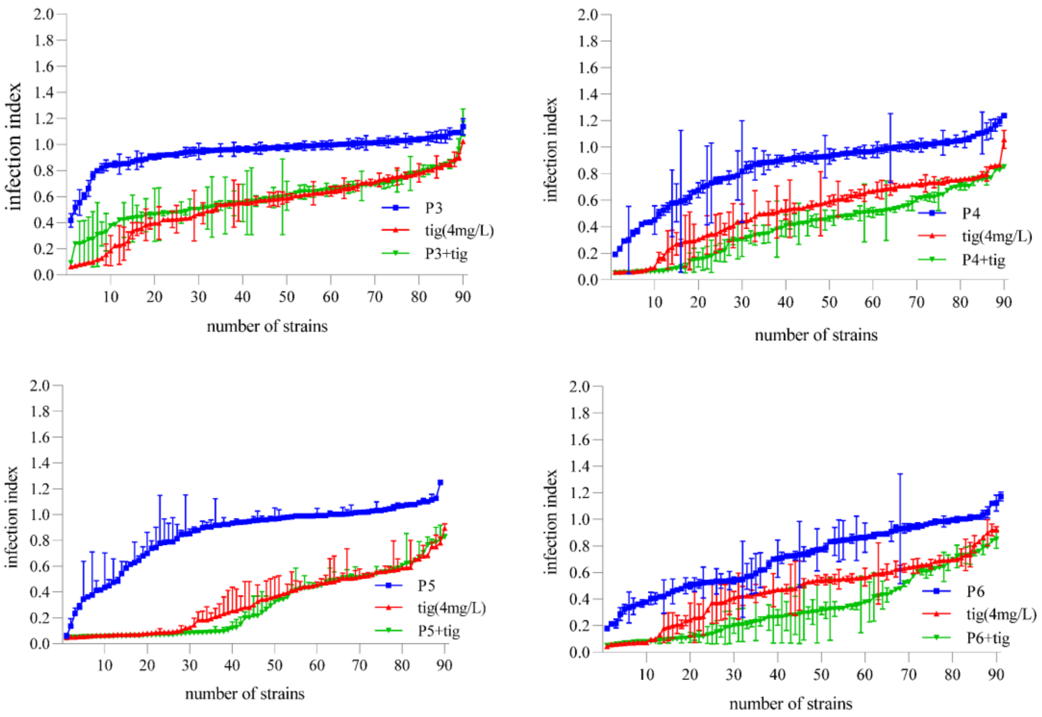


**Fig.S11 *In vitro* antimicrobial activity of four different phage cocktails (P3-P6) with or without tigecycline (4 mg/L) against 88 MDR Kp strains.** The infection index was indicated as the ratio of bacterial density between treated and non-treated groups ($OD_{600\text{-treated}}/OD_{600\text{-non-treated}}$). The threshold value for high bactericidal activity was 0.2, indicating the similar bactericidal effectiveness was observed in tigecycline and phage-tigecycline combinations. "P" denotes phage cocktails with the subsequent number indicating the number of phages used in the phage cocktails, for example, P3 indicates a cocktail composed of three different phages. Three independent replicates were performed for each treatment and the abbreviations for phage cocktail combinations were indicated in main Fig.3a.
